# Detrimental Effect of Type I IFNs During Acute Lung Infection With *Pseudomonas aeruginosa* Is Mediated Through the Stimulation of Neutrophil NETosis

**DOI:** 10.3389/fimmu.2019.02190

**Published:** 2019-09-11

**Authors:** Ekaterina Pylaeva, Sharareh Bordbari, Ilona Spyra, Anna Sophie Decker, Susanne Häussler, Vadim Vybornov, Stephan Lang, Jadwiga Jablonska

**Affiliations:** ^1^Translational Oncology, Department of Otorhinolaryngology, University Hospital Essen, Essen, Germany; ^2^Molecular Bacteriology, Helmholtz Centre for Infection Research (HZI), Braunschweig, Germany; ^3^Institute for Astronomy and Astrophysics, Eberhard Karls University, Tübingen, Germany

**Keywords:** *Pseudomonas aeruginosa*, neutrophils, IFN-β, IFNAR, innate immunity, bacterial infection, NETs, biofilms

## Abstract

*Pseudomonas aeruginosa* is an opportunistic multidrug-resistant pathogen, able to grow in biofilms. It causes life-threatening complications in diseases characterized by the up-regulation of type I interferon (IFN) signaling, such as cancer or viral infections. Since type I IFNs regulate multiple functions of neutrophils, which constitute the first line of anti-bacterial host defense, in this work we aimed to study how interferon-activated neutrophils influence the course of *P. aeruginosa* infection of the lung. In lungs of infected IFN-sufficient WT mice, significantly elevated bacteria load was observed, accompanied by the prominent lung tissue damage. At the same time IFN-deficient animals seem to be partly resistant to the infection. Lung neutrophils from such IFN-deficient animals release significantly lower amounts of neutrophil extracellular traps (NETs) and reactive oxygen species (ROS), as compared to WT neutrophils. Of note, such IFN-deficient neutrophils show significantly decreased capacity to stimulate biofilm formation by *P. aeruginosa*. Reduced biofilm production impairs in turn the survival of bacteria in a lung tissue. In line with that, treatment of neutrophils with recombinant IFN-β enhances their NETosis and stimulates biofilm formation by *Pseudomonas* after co-incubation with such neutrophils. Possibly, bacteria utilizes neutrophil-derived NETs as a scaffold for released biofilms. In agreement with this, *in vivo* treatment with ROS-scavengers, NETs disruption or usage of the bacterial strains unable to bind DNA, suppress neutrophil-mediated biofilm formation in the lungs. Together, our findings indicate that the excessive activation of neutrophils by type I IFNs leads to their boosted NETosis that in turn triggers biofilm formation by *P. aeruginosa* and supports its persistence in the infected lung. Targeting these mechanisms could offer a new therapeutic approach to prevent persistent bacterial infections in patients with diseases associated with the up-regulation of type I IFNs.

## Introduction

*Pseudomonas aeruginosa* is a gram-negative nosocomial pathogen. It shows relatively low virulence in healthy individuals, but can lead to life-threatening complications in hospitalized patients with cancer (1) or with virus infections (2). In such patients, *P. aeruginosa* infection often manifests as pneumonia, wound or implant infections, and sepsis. Besides the prolonged stay of such patients in the hospital, multiple invasive interventions, exposure to in-hospital microflora, declined tissue clearance, and immunological disorders may play a role in the pathogenesis of the infection (1). *P. aeruginosa* can form structured multicellular communities on surfaces, called biofilms. One of the components of biofilms is extracellular DNA (eDNA), which is attached by bacterial cationic exopolysaccharides due to its negative charge (3). Biofilm eDNA could be of bacterial or host cell origin (4–6). Hidden within self-produced matrix, bacteria in biofilms are protected from the host immune defense, antibiotics, or chemotherapy (7).

The host immune defense mechanisms against infection consist of structural barriers, soluble antimicrobial molecules, but also resident or recruited immune cells, such as neutrophils. The cogent role of neutrophil responses in acute *P. aeruginosa* infection of respiratory tract was proven by Koh et al. (8). Antibacterial properties of neutrophils include release of reactive oxygen species (ROS), production of bactericidal proteins, phagocytosis, and formation of neutrophil extracellular traps (NETosis) (9).

One of the potent regulators of neutrophil activity are type I interferons (IFNs) (10). These cytokines are released shortly after cell damage and are strong activators of the immune system (11). The up-regulation of type I IFN signaling is observed in various clinical conditions (12), including infections with *P. aeruginosa* (13). Type I IFNs are a large cytokine family including, among others, 14 IFN-α and a single IFN-β, all signaling through single receptor IFNAR (14). The master regulator of the whole IFN family is IFN-β (15). Type I IFNs modulate neutrophil phenotype and functions, e.g., by reducing their viability and migration, improving cytotoxicity and inhibiting their pro-angiogenic properties (16–18). During bacterial infections, the influence of type I IFNs on neutrophil functions appears to be controversial. In certain studies, the protective role of IFNs for the host was shown (19–24), while others revealed increased tissue damage and bacteria colonization in the presence of IFNs (25–29). At the same time, little is known about the influence of IFNs on neutrophil antibacterial functions during infection with *P. aeruginosa*.

In this manuscript, we set out to determine the role of type I IFNs in the modulation of neutrophil activity during acute lung infection with *P. aeruginosa* in mice and its impact on the course of infection. We have found that the absence of type I IFN signaling during acute phase of infection leads to reduced *Pseudomonas* persistence in the lung. Apparently, the absence of IFNs diminishes the capability of lung-associated neutrophils to release neutrophil extracellular traps (NETs). This in turn impairs biofilm formation by bacteria. Without protection that is provided by biofilms, *P. aeruginosa* is efficiently eliminated from the lungs of infected IFN-deficient mice, leading to the reduced bacterial load.

## Materials and Methods

### Bacteria

*Pseudomonas aeruginosa* strains that were used in this study: PA14 parental strain (wild-type serogroup O10 strain, cytotoxic ExoU+), *pelA* mutant PA14_24480 (*pelA* is the gene coding oligogalacturonide lyase related to exopolysaccharide production) and GFP PA14 *P. aeruginosa*. Bacteria have been cultured in Luria-Bertani (LB) broth for 3 h to reach the early exponential phase, washed twice in PBS, the optical density of 100 μl suspension was measured in 96 well flat-bottom cell culture plates (Cellstar, Greiner Bio One International GmbH, Frickenhausen, Germany) at 600 nm using a microplate reader Synergy 2 (BioTek Instruments, Inc., Vermont, U.S.). OD 0.4 corresponds to a bacterial density of 5 × 10^9^/ml, as determined by serial dilutions and colony-forming unit (CFU) assays. Bacteria concentration was adjusted to the desired values and verified by plating on 2% LB agar plates.

### Animals

Eight to twelve week-old mice of C56BL/6J wild type (WT), *Ifnar1*^−/−^ and *Ifnb1*^−/−^ strains were used in all experiments. Mice were bred and kept under SPF conditions in the animal facility of the University Hospital Essen (Germany).

### Lower Respiratory Tract Infection in Mice

For intratracheal inoculation of *P. aeruginosa*, mice were anesthetized with Ketamin (bela-pharm GmbH & Co, Vechta, Germany) 100 mg/kg and Xylazin (Ceva Tiergesundheit GmbH, Düsseldorf, Germany) 10 mg/kg in 0.9% NaCl solution, intubated and 2 × 10^6^ CFUs of *P. aeruginosa* in sterile PBS (50 μl) was administrated using the Minivent Mouse Ventilator type 845 (Harvard Apparatus, Massachusetts, U.S.) with stroke volume 150 μl and frequency 150 breaths/ min. The control of distribution of liquid in both lungs during intratracheal administration was performed prior to the experiments using Trypan blue (Sigma-Aldrich/Merck, Darmstadt, Germany). The adapted intratracheal method demonstrated accurate delivery and retention of *P. aeruginosa* in lungs. Animals were monitored post-operatively in a heated box until ambulant and clinically normal. Mice were transferred to a clean box with food and water *ad libitum* and monitored for 20 h. The duration of the experiment was chosen according to the published data confirming that biofilm formation by *P. aeruginosa* is a rapid process and takes place during first 10 h on plastic and 17 h in 3-D lung epithelial model (30, 31). After 20 h, mice were sacrificed. Heparinized blood was collected via heart puncture, plasma was prepared after centrifugation. Broncho-alveolar lavage (BAL) was collected after bronchial perfusion trough the trachea with 1 ml of sterile PBS. Lung tissue was mechanically homogenized in 1 ml PBS. BAL and lung homogenates were plated in serial dilutions to estimate CFUs on 2% LB agar and examined after 24 h incubation.

### Animal Treatment

*I.p*. injection *of* N-acetyl-L-cysteine (NAC) (Sigma-Aldrich/Merck, Darmstadt, Germany) treatment (dose 100 mg/kg) was performed 24 h and 1 h prior to *P. aeruginosa* inoculation.

*I.p*. treatment with rmIfnb (PBL assay science, Pestka Biomedical Laboratories, Inc, New Jersey, U.S.) in dose 1,000 U/mouse was performed 48 and 24 h before organs collection and neutrophil isolation.

### Assessment of Neutrophil Infiltration in Lungs During *P. aeruginosa* Infection

Lungs were collected as described above; organs from non-infected animals were used as a control. Lung tissue was digested using dispase 0.2 μg/ml, collagenase A 0.2 μg/ml, DNase I 100 μg/ml (all Sigma-Aldrich/Merck, Darmstadt, Germany) solution in DMEM (Gibco, Life Technologies/Thermo Fisher Scientific, Massachusetts, U.S.) containing 10% FCS and 1% penicillin-streptomycin). Cells were meshed through 50 μm filters (Cell Trics, Partec, Sysmex Europe GmbH, Goerlitz, Germany) and erythrocytes lysed in ACK buffer containing NH_4_Cl 150 mM, KHCO_3_ 10 mM, Na_2_EDTA 0.1 mM. BAL and single-cell suspensions were stained with antibodies listed below. The Ly6G^+^ neutrophil counts were assessed using BD FACS Canto system and data was analyzed using BD FACS Diva software (BD Biosciences, BD, New Jersey, U.S.).

### Isolation of Lung Neutrophils

For estimation of neutrophil functions, neutrophils were isolated from lungs of non-infected WT, *Ifnar1*^−/−^ and *Ifnb1*^−/−^ mice. Lung tissue was harvested from each animal under aseptic conditions; single cell suspension was prepared as described above. Single-cell suspensions were stained with antibodies listed below, CD11b^+^ Ly6G^+^ alive neutrophils were sorted using a FACS Aria cell sorter (BD Biosciences, BD, New Jersey, U.S.), and the purity of cells was assessed (≥95%) (Figure S1). After sorting cells were resuspended in DMEM containing 10% FCS.

### Antibodies

Anti-mouse CD16/32 (clone 2.4G2, BD Pharmingen, BD, New Jersey, U.S.), anti-Ly6G (clone 1A8, BD Pharmingen, BD, New Jersey, U.S.), anti-CD11b (clone M1/70, eBioscience, Affymetrix, California, U.S.). Viability Dye eFluor™ 780 (eBioscience, Affymetrix, California, U.S.) or DAPI (BioLegend, California, U.S.) were used to determine viable cells.

### NETs Release

Isolated neutrophils 15,000/well were incubated with *P. aeruginosa* (MOI 10) in glass-bottom 96-well plate (MatTek Corporation, Massachusetts, U.S.) pre-coated with poly-D-lysine 1 mg/ml (Sigma-Aldrich/Merck, Darmstadt, Germany) for 4 h at +37°C, 5% CO_2_, sterile medium was used as a negative control. Samples were fixed with paraformaldehyde (Thermo Fisher Scientific, Massachusetts, U.S.) to the final concentration 4%, permeabilized with Triton X-100 (Sigma-Aldrich/Merck, Darmstadt, Germany) 0.2% containing buffer. Since the visualization of NETs using DNA-intercalating dyes alone has the risk of detection of necrotic cells or the generation of artificial results based on dye-blocking peptides associated with NETs, antibody-based techniques are required to visualize NETs. Antibodies for citrullinated histones detect PAD4-dependent NETosis, but not NETs released through other mechanisms (32). Therefore, anti-histone 1 antibodies (Merck Millipore, Darmstadt, Germany) were used to detect all NETs. Donkey-anti-mouse-AF564 (Invitrogen, Thermo Fisher Scientific, Massachusetts, U.S.) were used as secondary antibodies. Stainings were mounted with ProLong Gold Antifade Mountant with DAPI (Invitrogen, Thermo Fisher Scientific, Massachusetts, U.S.). Percent of NET-producing cells and NETs length were estimated by microscopy.

### Reactive Oxygen Species

Lung tissue was harvested from non-infected WT, *Ifnb1*^−/−^ and *Ifnar1*^−/−^ animals under aseptic conditions; single cell suspensions were prepared and stained with antibodies as described above. Cells were washed and resuspended in DMEM containing 10% FCS, *P. aeruginosa* PA14 WT MOI 10 was added. Sterile medium was used as negative control. ROS production by Ly6G^+^ alive neutrophils was estimated after 60 min of exposure to *P. aeruginosa* using Dihydrorhodamine 123 (Sigma-Aldrich/Merck, Darmstadt, Germany) with flow cytometry.

### Phagocytosis

Lung tissue was harvested from non-infected WT and *Ifnar1*^−/−^ animals under aseptic conditions; single cell suspension was prepared and stained with antibodies. Cells were then washed and resuspended in DMEM containing 10% FCS, *P. aeruginosa* PA14 WT-GFP (MOI 10) added. Phagocytosis of GFP-bacteria by Ly6G^+^ neutrophils was estimated after 60 min using flow cytometry.

### Endocytosis and Procession in Phagolysosomes

Lung tissue was harvested from non-infected WT and *Ifnar1*^−/−^ animals; single cell suspension prepared and stained with antibodies. Cells were washed and resuspended in DMEM containing 10% FCS, DQ ovalbumin added (Molecular probes, Invitrogen, Thermo Fisher Scientific, Massachusetts, U.S.). After 30 min, the reaction was stopped by placing the plate on ice, green fluorescence of processed DQ ovalbumin in Ly6G^+^ neutrophils was estimated using flow cytometry.

### Migration to Lipopolysaccharide (LPS)

Cell migration was evaluated using a two-chamber transwell system (3 μm pore size) cell culture inserts (Falcon, Corning, New York, U.S.). Seven hundred microliter of DMEM + 10% FCS containing LPS (Invitrogen, Thermo Fisher Scientific, Massachusetts, U.S.) in concentration 1 ng/ml or LPS-free medium, as a negative control for spontaneous migration, were added to the lower chamber. Isolated lung WT and *Ifnar1*^−/−^ neutrophils 3 × 10^5^ cells in 300 μl DMEM + 10% FCS were added to the upper chamber, then the chamber was placed into medium for 3 h in an incubator at 37°C and 5% CO_2_. Cells transmigrated to the lower chamber were counted using CASY (Innovatis, Roche Innovatis AG, Bielefeld, Deutschland). Measurements were performed in independent experiments and mean counts calculated.

### Biofilms

96-well flat plastic-bottom plates were used, lung neutrophils in concentration 2 × 10^5^/well and *P. aeruginosa* (MOI 10) were added to the final volume 150 μl. After 72 h wells were washed with deionized water twice and stained with 0.4% crystal violet (Sigma-Aldrich/Merck, Darmstadt, Germany), then washed with deionized water twice. Crystal violet is a basic protein dye that stains negatively charged surface molecules of viable and alive cells and extracellular matrix of polysaccharides (33). Microscopy photographs of dry wells were taken using AMD EVOS fl digital inverted microscope in brightfield. Crystal violet stain was measured after the addition of 30% acetic acid on a plate reader at OD600. All samples were tested at least in 3 independent wells.

For fluorescent staining biofilms of *P. aeruginosa*-GFP were similarly prepared in glass-bottom non-covered 96-well plates, permeabilized with TritonX 0.2%, stained with anti-histone 1 antibodies and donkey-anti-mouse-AF564 secondary antibodies, mounted with DAPI ProLong Gold Antifade Mountant. No DAPI signal from nuclei was detected, proving the absence of alive neutrophils with preserved nuclei in 72 h co-culture.

Concentration of DNase in *in vitro* studies: 100 μg/ ml, NAC concentration: 100 μM.

### Histology

For histological examination of lungs, WT, *Ifnb1*^−/−^, and *Ifnar1*^−/−^ mice were infected *i.t*. with *P. aeruginosa*. At the certain time point mice were sacrificed, lungs perfused with Tissue-Tek O.C.T. Compound (Sakura Finetek, Japan) containing 5% paraformaldehyde, the lumen of the trachea was fixed with ligature; lungs were dissected and snap frozen at −80°C. 7-μm cryosections were fixed with ice-cold acetone, stained with hematoxylin-eosin or anti-histone 1 and DAPI, dried and mounted with Neo-Mount (Merck, Darmstadt, Germany).

### Microscopy

Microscopy was performed using Zeiss AxioObserver.Z1 Inverted Microscope with ApoTome Optical Sectioning equipped with filters for: DAPI, FITC, Alexa Fluor 488, GFP, DsRed, Cy3 or Olympus BX51 upright epifluorescence microscope. Images were processed with ZEN Blue 2012 software or CellSens Dimension software (Olympus), respectively, and analyzed with ImageJ.

### ELISA

TNF-α in plasma samples were analyzed with ELISA (R&D Systems, Minnesota, U.S.) according to manufacturer protocols.

### RT-qPCR

RNA was isolated from WT, *Ifnar1*^−/−^, and *Ifnb1*^−/−^ neutrophils, using the RNeasy Mini Kit (Qiagen, Venlo, Netherlands) and cDNA prepared. Real-time RT-PCR was performed using primers listed in Table 1.

**Table 1:** Primers used for qRT-PCR.

**Gene**	**Forward (5^**′**^ to 3^**′**^)**	**Reverse (5^**′**^ to 3^**′**^)**
*Mpo*	CGTGTCAAGTGGCTGTGCCTAT	AACCAGCGTACAAAGGCACGGT
*Lyz2*	TGCCAGAACTCTGAAAAGGAATGG	CAGTGCTTTGGTCTCCACGGTT
*Def1*	AACTGAGGAGCAGCCAGGAGAA	CTTCCTTTGCAGCCTCTTGATCT
*Nox2*	TGGCGATCTCAGCAAAAGGTGG	GTACTGTCCCACCTCCATCTTG
*Rps9*	TTGACGCTAGACGAGAAGGAT	AATCCAGCTTCATCTTGCCCT

### Expression of IFNAR Subunits on Circulating Leukocytes in Lung Cancer and Lower Respiratory Tract (LRT) Viral Infection

We used previously published microarray data deposited in the Gene Expression Omnibus database GSE42834 (34) and GSE60244 (35). Analyzed samples are listed in Supplementary Materials.

### Statistics

Statistical analyses were performed using Kruskal-Wallis ANOVA for multiple comparisons with the Bonferroni correction, and Mann-Whitney U-test for two independent samples. *P* < 0.05 was considered significant.

### Study Approval

The animal experiments have been approved by the regulatory authorities LANUV (Das Landesamt für Natur, Umwelt und Verbraucherschutz Nordrhein-Westfalen), Germany. Our animal care and use protocols adhere to the regulations of das Deutsche Tierschutzgesetz (TierSchG) and follow FELASA recommendations.

## Results

### Elevated Expression of IFNAR on Leukocytes From Patients With Cancer and Viral Infections

Diseases such as cancer or viral infections are characterized with the enhanced production of type I IFNs (12). To study the regulation of type I IFN receptor (IFNAR) on immune cells, we performed the analysis of available Gene Expression Omnibus databases GSE60244 and GSE42834 (34, 35). We observed an up-regulation of IFNAR subunits 1 and 2 on circulating leukocytes during cancer and viral infections, giving the evidence of the activated interferon signaling in immune cells during the course of disease (Table 2).

**Table 2:** Expression of IFNAR subunits on circulating leukocytes in lung cancer and lower respiratory tract (LRT) viral infection.

	**LUNG CANCER Healthy vs. Disease**	**VIRAL LRT INFECTION Healthy vs. Disease**
*IFNAR1*	−0.2 (−0.4; 0.0) vs. 0.6 (0.4; 0.8) *p* < 0.0001	278 (221; 347) vs. 382 (319; 534) *p* < 0.0001
*IFNAR2*	−0.2 (−0.5; 0.1) vs. 0.2 (−0.0; 0.4) *p* < 0.0001	354 (316; 383) vs. 381 (316; 480) *p* < 0.05

*To study the regulation of type I IFN receptor (IFNAR) on immune cells, we performed the analysis of available Gene Expression Omnibus databases GSE60244 and GSE42834 (34, 35). Data are shown as median (interquartile range)*.

Such a significant up-regulation of IFNAR on leukocytes in diseases commonly associated with *P. aeruginosa* infections (e.g., lung cancer, viral lower respiratory tract infections) (1, 2), prompted us to evaluate the role of type I IFNs in the regulation of antibacterial functions of neutrophils in the model of *P. aeruginosa* induced pneumonia.

### IFN-Deficient Mice Infected With *P. aeruginosa* Show Lower Lung Colonization and Reduced Tissue Damage, as Compared to WT Animals

**Figure 1:**
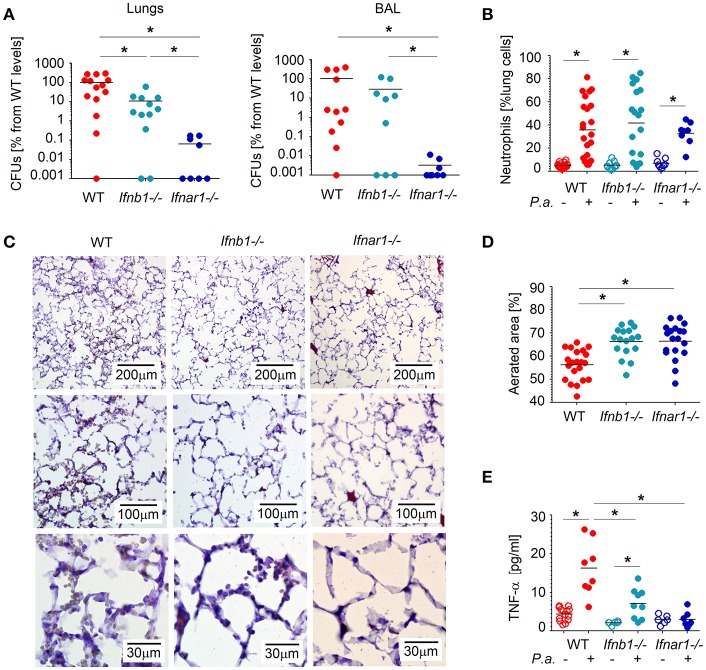
Type I IFNs contribute to tissue damage and *P. aeruginosa* survival in lungs. **(A)** Elevated bacteria load in lungs and in broncho-alveolar lavage (BAL) of WT mice. WT, *Ifnb1*^−/−^ and *Ifnar1*^−/−^ mice were infected with *P. aeruginosa* and sacrificed after 20 h. Bacterial load in organs was determined by serial dilutions and plating on LB agar, CFUs were determined. At least 4 animals per group in 2 independent experiments were used. **(B)** Elevated infiltration of neutrophils into lungs of WT mice. Neutrophils in lungs were determined by flow cytometry. **(C,D)** Better aerated lungs in IFN-deficient mice after *Pseudomonas* infection. Five micrometer lung cryosections were stained with hematoxylin-eosin, scale bars: 200, 100, and 30 μm. At least 5 fields of view in 4 lungs per group were analyzed. **(E)** Systemic TNF-α levels before and after infection were measured in plasma with ELISA. *P.a., Pseudomonas aeruginosa*. Data are shown as individual values and mean, *p < 0.05.

*P. aeruginosa* often cause infections of lower respiratory tract and is associated with elevated type I IFN signaling in the host (13). Therefore, we were interested of how IFN signaling influence the course of *Pseudomonas* infection. To this end, we infected mice intratracheally with *P. aeruginosa* PA14, and compared the course of infection between WT and IFN-deficient *(Ifnar1*^−/−^ and *Ifnb1*^−/−^) animals. We observed that after 20 h *P. aeruginosa* was efficiently cleared from the lung and BAL of IFN-deficient mice, while WT mice showed elevated bacteria counts (Figure 1A).

Since neutrophils are the first line of defense and play an important role in controlling bacterial infections, we were interested if the different susceptibility for *Pseudomonas* infection between WT and IFN-deficient animals could be due to altered neutrophil counts in infected lungs. Therefore, we evaluated numbers of Ly6G^+^ neutrophils in the lungs of WT and IFN-deficient animals (gating strategy see Figure S1), but no significant differences between the mouse strains were observed (Figure 1B).

As neutrophil counts obviously do not contribute to elevated susceptibility of WT mice to *Pseudomonas* infection, we were interested if the activation status of these cells could be different between tested mouse strains. Activated neutrophils may lead to tissue damage; therefore, we analyzed structural changes, such as swelling of alveoli walls or reduced aeration, in the lungs of mice infected with *P. aeruginosa*. Importantly, we could observe more prominent tissue damage in the lungs of WT mice, as compared to IFN-deficient animals (Figures 1C,D). In line with this, systemic level of TNF-α, which is the key component of inflammatory responses, was significantly elevated in WT animals (Figure 1E).

Together, these findings suggest that altered neutrophil activity, but not their counts, are responsible for the observed sensitivity of WT mice to *P. aeruginosa* infection.

### WT Lung Neutrophils Release Elevated Levels of Reactive Oxygen Species and Neutrophil Extracellular Traps in Response to *Pseudomonas* Infection

**Figure 2:**
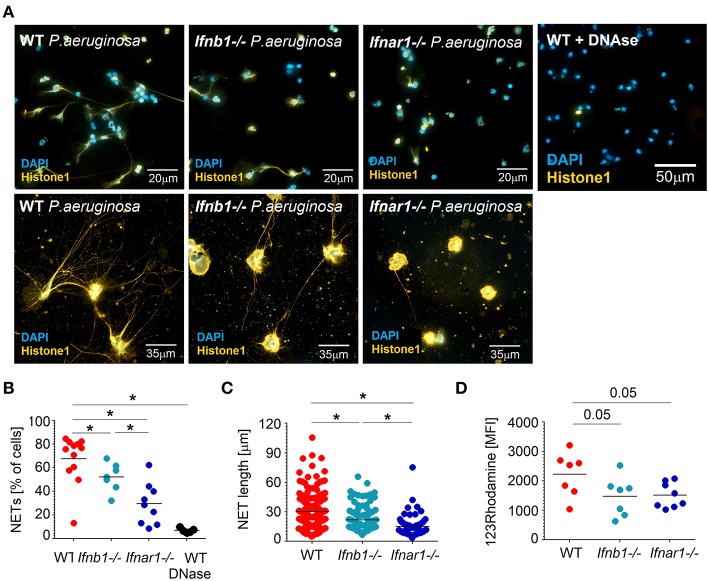
Lower ROS production and NET formation in IFN-deficient neutrophils after infection with *P. aeruginosa*. **(A–C)** Reduced NETosis in IFN-deficient mice after infection with *P. aeruginosa*. Lung neutrophils isolated from WT, *Ifnb1*^−/−^ and *Ifnar1*^−/−^ mice were challenged with *P. aeruginosa* MOI 10 for 4 h, NETs were stained with anti-histone 1 (orange) and DAPI (blue). Exemplified staining of NETs **(A)**, scale bars: 20 and 13 μm. Quantification of NET-positive cells **(B)** and NET length **(C)**. At least 9 fields of view per group were analyzed. Treatment with DNase proves the major role of DNA as a component of NETs. **(D)** ROS production in presence of *P. aeruginosa* is higher in WT mice. Single cell lung suspension from WT, *Ifnb1*^−/−^ and *Ifnar1*^−/−^ mice was challenged with *P. aeruginosa* MOI 10 for 1 h, stained with 123Rhodamine and released ROS evaluated in neutrophils using flow cytometry. At least 7 animals per group were included. Data are shown as individual values and mean, *p < 0.05.

As neutrophils are the major antibacterial effector cells during *P. aeruginosa* infection and their activation seem to be altered in IFN-deficient mice, we aimed to characterize antibacterial functions of neutrophils isolated from both mouse strains. For this purpose, we challenged isolated mouse lung neutrophils (>95% purity, Figure S1) with *P. aeruginosa* MOI 10. Notably, significantly impaired capacity to release NETs was observed in IFN-deficient neutrophils, as compared to WT (Figures 2A,B). Moreover, the ability to produce long NETs was also significantly reduced in such neutrophils (Figures 2A–C).

Reactive oxygen species (ROS) are involved in direct cytotoxicity of neutrophils, but are also suggested to trigger the NET release by these cells (36). Therefore, we measured the production of ROS by lung-associated neutrophils in response to *P. aeruginosa* infection and compared WT with IFN-deficient mice. We could observe that IFN-deficient neutrophils have lower capability to produce ROS in these conditions (Figure 2D).

Subsequently, we estimated migration, phagocytosis, endocytosis and phagolysosomal degradation in neutrophils infected with *P. aeruginosa*. Moreover, we assessed the expression of key antibacterial genes in such neutrophils, and compared WT and IFN-deficient animals. IFN-deficient neutrophils showed significantly higher migratory capacity toward bacterial lipopolysaccharide (LPS) (Figure S2A). Other functions of neutrophils, such as phagocytosis (Figure S2B), endocytosis and phagolysosomal processing (Figure S2C) were not significantly different between WT and IFN-deficient mice. Furthermore, the expression of molecules responsible for antibacterial functions of neutrophils, such as defensins, lysozyme, myeloperoxidase, NADPH-oxidase, was not significantly altered between WT and IFN-deficient neutrophils (Figure S2D).

Altogether, we observed that WT neutrophils release more ROS and NETs in response to *Pseudomonas* infection, while other antibacterial functions of neutrophils are not significantly altered. Elevated ROS and NETs are probably responsible for prominent tissue damage that is observed in the lungs of infected WT mice.

### WT Neutrophils Induce Strong Biofilm Formation by *P. aeruginosa*

**Figure 3:**
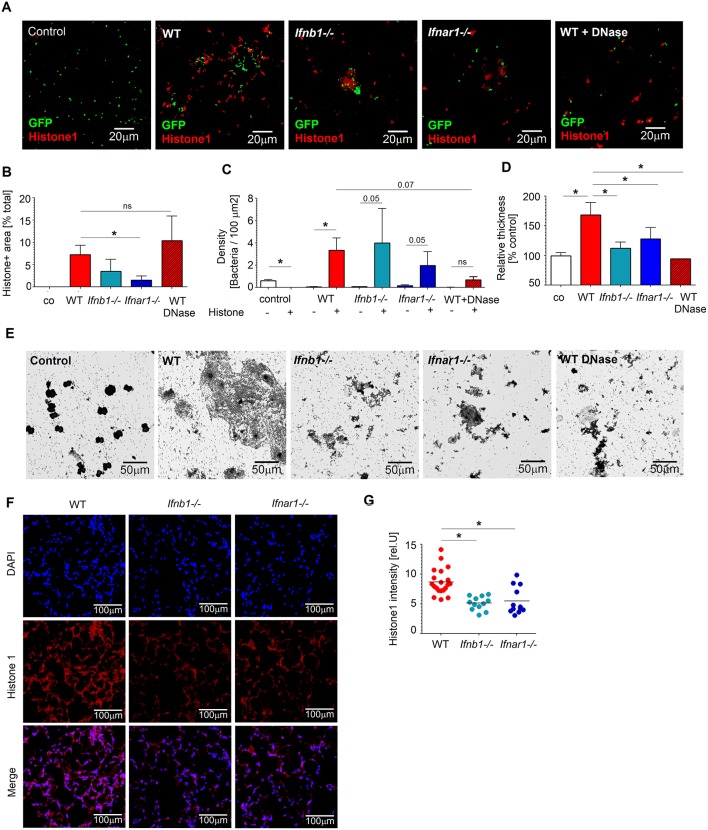
IFN-sufficient neutrophils support biofilm formation *in vitro* and *in vivo* after *Pseudomonas* infection. **(A–C)** Higher numbers of bacteria associated with histone-rich areas after *in vitro* infection of WT lung neutrophils. Lung neutrophils isolated from WT, *Ifnb1*^−/−^ and *Ifnar1*^−/−^ mice were challenged with *P. aeruginosa* GFP MOI 10 for 3 days, wells were stained for histone 1 (red). Control—bacteria without neutrophils; Exemplified staining, scale bars: 20 μm **(A)**. Quantification of histone1-positive areas **(B)** and *P. aeruginosa* accumulation in histone 1-rich vs. histone 1-negative areas **(C)**. Quantification was done in triplicate with at least 5 fields of view per well. **(D,E)** Elevated biofilm formation after incubation of WT neutrophils with *P. aeruginosa*. Isolated lung neutrophils were challenged with bacteria MOI 10 for 3 days, biofilms were stained with crystal violet, control—bacteria alone without neutrophils. Exemplified biofilm staining, scale bars: 50 μm **(D)**. Quantification of biofilms was performed using acetic acid and OD measurements **(E)**. Data are shown as mean ± SEM, *p < 0.05. **(F,G)** Elevated accumulation of neutrophil-derived NETs (histone 1^+^) in lungs of WT mice after *P. aeruginosa* infection. WT, *Ifnb1*^−/−^ and *Ifnar1*^−/−^ mice were *i.t*. infected, sacrificed after 20 h, 5 μm cryosections of lungs were stained with anti-histone 1 (red) and DAPI (blue). Exemplified lung staining, scale bars: 100 μm **(F)**, Quantification of the histone 1 intensity in lungs was analyzed in 4–5 fields of view in 4 lungs per group. Data are shown as individual values and mean, *p < 0.05.

WT mice show higher tissue damage after *Pseudomonas* infection, but also elevated bacteria count in lung. Since WT neutrophils release higher amounts of ROS and NETs, we were interested if this influences also the control of bacteria by these cells. Therefore, we co-incubated *P. aeruginosa* with isolated WT and IFN-deficient lung neutrophils, and assessed survival of bacteria. In agreement with our previous findings, neutrophils released high amount of NETs (histone 1^+^) in response to *Pseudomonas*. This was significantly higher in WT neutrophils, compared to IFN-deficient neutrophils (Figures 3A,B). Of note, co-incubation of *P. aeruginosa* with neutrophils resulted in significantly elevated survival of bacteria, compared to bacteria alone (control). Moreover, *Pseudomonas* were mainly found in NETs (Figures 3A,C).

To validate the role of NETs in this experimental setting, we disrupted them using DNase and estimated *P. aeruginosa* counts. Interestingly, treatment with DNase reduced survival of bacteria (Figures 3A–C), suggesting an importance of NETs for *Pseudomonas* persistence.

It is known that *Pseudomonas* survival in tissues depends strongly on the ability of this bacterium to form biofilms. Therefore, we assessed biofilm development after co-incubation of bacteria with lung neutrophils and observed significantly elevated production of biofilms in the presence of neutrophils, as compared to bacteria alone. Moreover, WT neutrophils seemed to be more efficient stimulators of biofilm formation than IFN-deficient neutrophils. Since biofilm formation by *Pseudomonas* correlates with higher NETs release from neutrophils, we tested biofilm production by bacteria in the presence of neutrophils treated with DNase. Staining with crystal violet proved diminished biofilm formation in this experimental setting (Figures 3D,E).

We hypothesized that NETs released from neutrophils trigger *Pseudomonas* biofilm formation. This in turn should support the persistence of bacteria in lungs of infected mice. To test this *in vivo*, we infected mice with *P. aeruginosa* and stained their lungs to visualize NETs components. In agreement with our *in vitro* data, we observed higher amounts of histone1 positive NETs in lungs of infected WT mice, as compared to IFN-deficient *Ifnb1*^−/−^ or *Ifnar1*^−/−^ animals (Figures 3F,G). Higher NETs presence in lungs of WT mice correlated positively with elevated bacteria counts.

Thus, upon infection with *P. aeruginosa*, NETs that are released from lung-associated neutrophils support formation of biofilms by the bacterium. Biofilms support *Pseudomonas* persistence in the lung, by protecting it from the immune system. This phenomenon is responsible for the observed elevated counts of bacteria in lungs of WT mice, as compared with IFN-deficient animals.

### rmIFN-β Treatment Supports NETs Formation by Neutrophils

**Figure 4:**
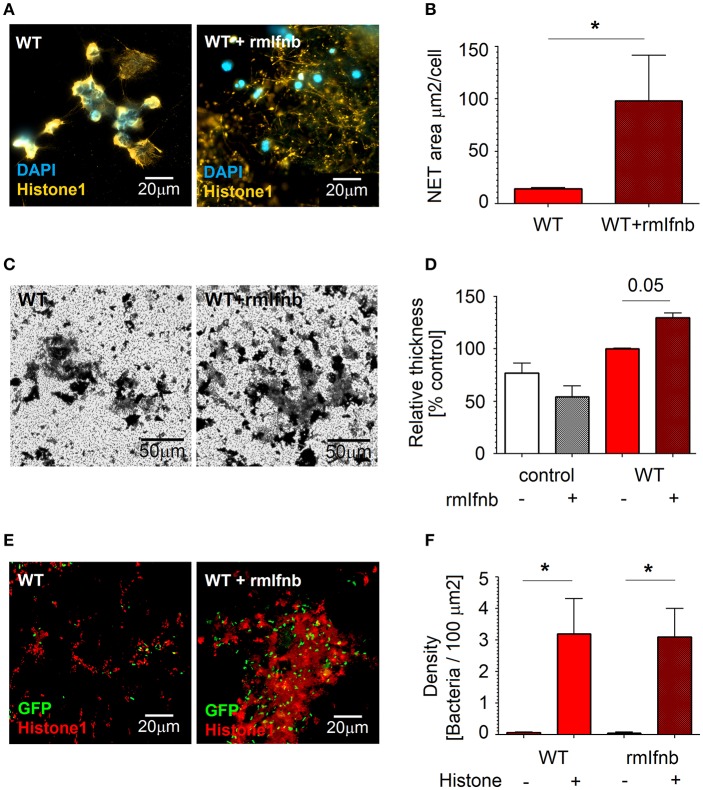
rmIfnb stimulates NETs release by neutrophils and supports biofilm formation. **(A,B)** NETosis is enhanced by rmIFNβ. Lung neutrophils were isolated from untreated and rmIfnb-treated WT mice. Cells were challenged with *P. aeruginosa* MOI 10 for 4 h, NETs were stained with anti-histone 1 (orange) and DAPI (blue). Exemplified staining, scale bars: 20 μm. **(A)** Quantification of the area covered with NETs. **(B)** At least 5 fields of view per group were analyzed. **(C,D)** Elevated biofilm production after IFN treatment. Lung neutrophils isolated from untreated and rmIFNβ-treated WT mice were challenged with *P. aeruginosa* MOI 10 for 3 days, biofilms were stained with crystal violet. Exemplified staining, scale bars: 50 μm. **(C)** Quantification of biofilms was performed after dilution with acetic acid and OD measurement. **(D)** Data are shown as mean ± SEM, *p < 0.05. **(E,F)** Higher numbers of bacteria associated with histone-rich areas after *in vitro* infection of WT lung neutrophils. Lung neutrophils isolated from WT naive and IFN-β-treated animals were challenged with *P. aeruginosa* GFP MOI 10 for 3 days, wells were stained for histone 1 (red). Exemplified staining, scale bars: 20μm **(E)**. Quantification of *P. aeruginosa* accumulation in histone 1-rich vs. histone 1-negative areas **(F)**.

To proof that enhanced NETs release is due to IFN availability, we treated mice with 1,000 U of rmIFN-β for 3 days, isolated lung neutrophils and estimated NETs formation in response to *Pseudomonas* infection. Indeed, we could observe significantly elevated NETs release upon rmIFN-β treatment (Figures 4A,B). Interestingly, also the structure of produced NETs was altered (Figure 4A). In agreement with our hypothesis that NETs stimulate biofilm production by *P. aeruginosa*, elevated biofilm formation was observed after co-incubation of bacteria with IFN-β pretreated neutrophils (Figures 4C,D). High numbers of bacteria associated with histone-rich areas after IFN-β treatment were proven (Figures 4E,F).

Thus, IFNs stimulate lung-associated neutrophils to release NETs during *P. aeruginosa* infection. This in turn triggers the formation of bacterial biofilms.

### NET-Derived DNA Is Essential for *P. aeruginosa* Biofilm Formation

**Figure 5:**
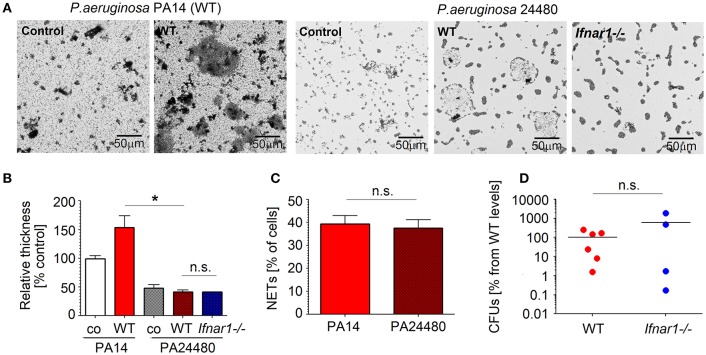
NETs released by neutrophils are important components of *P. aeruginosa* biofilms. **(A,B)** *Pseudomonas pelA* mutant strain 24480 which is unable bind to eDNA shows reduced biofilm formation. Neutrophils isolated from WT mice were challenged with *P. aeruginosa* PA14 and 24480 strain at MOI 10 for 3 days. Biofilms were stained with crystal violet, control—bacteria only. Exemplified biofilm staining, scale bars: 50 μm. **(A)** Quantification of biofilms was performed using acetic acid and OD measurement **(B)**. **(C)** Comparable NET formation in response to different *P. aeruginosa* strains: PA14 and 24480 strain. Neutrophils isolated from WT mice were challenged with *P. aeruginosa* PA14 and 24480 strains at MOI 10 for 4 h, NETs were stained with anti-histone 1 and DAPI, the number of NET-positive cells was quantified. At least 5 fields of view per group were analyzed. Data are shown as mean ± SEM, *p < 0.05. **(D)** Inhibited biofilm production by *pelA Pseudomonas* mutant abrogated differences in bacteria clearance between infected WT and *Ifnar1*^−/−^ mice. WT and *Ifnar1*^−/−^ mice were infected with 24480 strain, animals were sacrificed after 20 h, bacterial load in organs was determined by serial dilutions and plating on LB agar, CFUs were calculated. At least 4 animals per group were included. Data are shown as individual values and mean, *p < 0.05.

To prove the involvement of NETs in biofilm formation, *P. aeruginosa pelA* mutant strain PA24480 was used. Pel is cationic exopolysaccharide that cross-links extracellular DNA to the biofilm matrix (37), therefore PA24480 mutant (lacking Pel exopolysaccharide) is unable to bind to the extracellular NETs DNA. We isolated lung neutrophils and co-cultured them with *Pseudomonas* PA24480. NETs release and biofilm formation was evaluated. We could observe that biofilm formation by *Pseudomonas* PA24480 was significantly inhibited for both WT and IFN-deficient neutrophils (Figures 5A,B). Importantly, the capacity to induce NETs was comparable between PA24480 and wild type PA14 strain (Figure 5C). Therefore, we assume that the inhibition of biofilm formation by PA24480 strain is solely due to its inability to bind NETs DNA.

To corroborate this *in vivo*, we infected WT and IFN-deficient mice with *Pseudomonas* PA24480, and compared the bacterial load in lungs after 20 h. As expected, we could not observe any significant differences in bacterial load between WT and IFN-deficient mice (Figure 5D). Devoid of biofilm protection, *P. aeruginosa* in WT mice had no advantage over those in IFN-deficient mice.

Taken together, binding of bacteria to DNA in NETs is an essential step facilitating the production of biofilms by *P. aeruginosa* and supporting its persistence in infected lungs.

### N-Acetylcysteine Prevents NET-Dependent Biofilm Formation by *P. aeruginosa* and Leads to Effective Bacteria Elimination From the Lung

We could demonstrate that NET-dependent biofilm formation supports *P. aeruginosa*-persistence in infected lungs. Since production of ROS by neutrophils is one of the triggers for NETs formation, the reduction of ROS may provide therapeutic solution for persistent *Pseudomonas* lung infections. One of the factors that are known to efficiently reduce ROS production in neutrophils is N-acetylcysteine (NAC) (38). Here, we set out to determine the effect of NAC on NETs formation and consequential biofilm development by *P. aeruginosa*.

**Figure 6:**
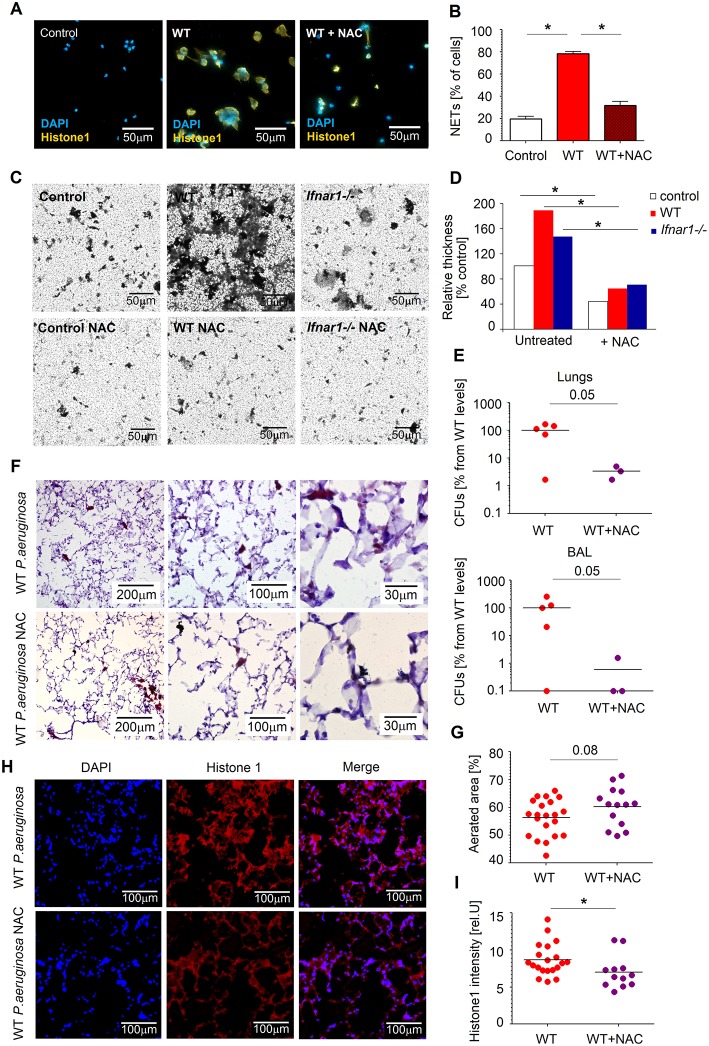
NAC disrupts NETosis and prevents biofilm formation. **(A,B)** NAC prevents NETs formation by WT neutrophils. Neutrophils were challenged with *P. aeruginosa* MOI 10 for 4 h in presence and absence of NAC, NETs were stained with anti-histone 1 (orange) and DAPI (blue). Exemplified staining, scale bars: 50 μm **(A)**. Quantification of NET-positive cells **(B)**. At least 5 fields of view per group were analyzed. **(C,D)** NAC treatment of neutrophils and following inhibition of NETosis prevents biofilm formation by *Pseudomonas*. Neutrophils were challenged with *P. aeruginosa* as described above (+ and –NAC), biofilms were stained with crystal violet, control—bacteria only. Exemplified biofilm staining, scale bars: 50 μm. **(C)** Quantification of biofilms was performed using acetic acid and OD measurement. **(D)** Data are shown as mean ± SEM, *p < 0.05. **(E)** Reduced bacteria load in lungs of NAC-treated mice. WT mice were treated with NAC at day−1 and 1 h before *Pseudomonas* infection. Mice were sacrificed after 20 h, bacterial load in lungs and BAL determined by plating of serial dilutions on LB agar. CFUs were estimated. At least 3 animals per group were included. **(F,G)** Less lung injury after NAC treatment of mice. Mice were infected as described above in E, lungs were isolated, 5 μm cryosections prepared and stained with hematoxylin-eosin. Representative staining is shown, scale bars: 200, 100, and 30 μm **(F)**, Quantification of aerated areas in at least 5 fields of view in 4 lungs per group **(G)**. **(H,I)** Reduced NETs content in lungs of mice treated with NAC. WT mice were infected as described above, animals were sacrificed after 20 h, 5 μm cryosections of lungs were stained with anti-histone 1 (red) and DAPI (blue). Representative staining of NETs in lungs is shown, scale bars: 100 μm **(H)**, Quantification of NETs in lungs in 4–5 fields of view in 4 lungs per group **(I)**. Data are shown as individual values and mean, *p < 0.05.

First, we isolated WT lung neutrophils, co-incubated them with bacteria and quantified NETs release and biofilm production in presence vs. absence of NAC. Importantly, treatment of neutrophils with NAC led to significant suppression of NETs release (Figures 6A,B). In agreement, biofilm formation by *P. aeruginosa* was also diminished in this experimental setting (Figures 6C,D).

Next, we have evaluated therapeutic effectiveness of NAC in preventing *Pseudomonas* persistence in lungs of infected mice. We treated WT mice with NAC and infected them with *Pseudomonas*. Notably, we could observe reduced counts of bacteria in the lungs and BAL of mice treated with NAC, as compared to untreated animals (Figure 6E). NAC-treated animals showed also diminished lung tissue damage (preserved aeration areas) (Figures 6F,G) and lower amounts of NET components (histone 1) (Figures 6H,I).

Thus, prevention of NET release by NAC leads to reduced survival of bacteria in lungs and at the same time to lower lung tissue damage. Therefore, NAC treatment may provide a potent tool in prophylaxis and treatment of persistent *P. aeruginosa* infections.

In sum, in this manuscript we could demonstrate that during *Pseudomonas aeruginosa* infection type I IFNs excessively activate neutrophils to release NETs. Binding to such NETs triggers formation of biofilms by *P. aeruginosa*. Accumulating biofilms provide in turn a protective niche that supports bacteria persistence in lungs. Therefore, NETs disrupting agents should be considered as anti-bacterial treatment and prophylaxis in diseases associated with type I IFN up-regulation.

## Discussion

Viral infections or cancer are associated with the upregulation of type I IFNs and IFN-dependent immune responses, as well as with the enhanced rate of bacterial complications (1, 2). Moreover, infections with bacteria, such as *P. aeruginosa*, are often associated with the elevated type I IFN signaling in lung epithelial cells (13). The data concerning the impact of high IFN levels on the bactericidal functions of neutrophils are limited. Clinical observations show that neutrophils isolated from patients with elevated type I IFN levels, spontaneously produced NETs and displayed indicators of oxidative and mitochondrial stress (39). A similar phenomenon was observed in neutrophils from healthy controls that were exposed to patient plasma samples or exogeneous IFN (39).

Here, we demonstrate that type I IFN-mediated activation of neutrophils in lungs may lead not only to the prominent tissue damage, but it can also support biofilm formation by *Pseudomonas* and its tissue persistence.

The role of neutrophils during *P. aeruginosa* infections is not clear. The high (up to 50%) proportion of the neutrophils in the body exists in so called marginating pool in the microcirculatory vessels of the liver, spleen, bone marrow and lungs, and can be fast mobilized in response to infection. The consumption of neutrophils at the sites of inflammation is compensated by the elevated emergency myelopoiesis (40). Neutrophils rapidly respond to the infection, the release of NETs by these cells can be observed already during first 10 min after contact with the pathogen (41).

*Pseudomonas aeruginosa* evolved multiple mechanisms to evade host immune responses. For example, ROS produced by neutrophils triggers mutations in *P. aeruginosa*, including those responsible for exopolysaccharide production and biofilm formation (42). This may promote further adaptation and survival of bacteria. Biofilm formation by *P. aeruginosa* could be supported by eDNA released by neutrophils (5). Bacteria within biofilms are protected from the immune system and eventual antibiotic treatment (4, 6), which leads to their high persistence in patients. In agreement, we observe the accumulation of GFP-expressing (alive) bacteria in histone-rich areas (NETs). Obviously, *Pseudomonas* is not only able to survive the contact with neutrophil NETs, but can also utilize NETs components to produce biofilms.

Persistence of the pathogen leads to elevated immune responses and severe cytotoxicity. Excessive activation of neutrophils, or their prolonged survival in tissues during chronic inflammation, is associated with a high tissue damage due to release of proteases, such as neutrophil elastase (43) or matrix metallopeptidase-9 (44). Resulting cleavage of opsonizing complement molecules or receptors on immune cells could lead to immune mismatch and thus to the escape from the immune control (45, 46).

We could demonstrate that type I IFNs excessively activate neutrophils and trigger their ROS production and NETs release in response to *P. aeruginosa* infection. This supports biofilm formation by the bacteria and their survival in lungs. Interestingly, the effect of type I IFNs varies between different models of bacterial, fungal and parasite infections. In certain studies the protective role of IFNs for the host was proven during the microbial infections (19–24), while others revealed increased tissue damage and bacteria colonization in the presence of IFNs (25–29). These differences might be partly explained by the involvement of the distinct cell subsets in antibacterial immune responses, which is due to the diverse, extra- or intracellular, localization of pathogens. Moreover, bacteria developed various protecting mechanisms, such as biofilm formation, that are influencing the outcome of infection. Numerous bacterial species, including *P. aeruginosa*, utilize extracellular eDNA for the biofilm matrix formation (3). Possibly, the mechanism responsible for it includes surface modifications that protect from DNA-induced membrane destabilization and NET-mediated killing (47). Another host-driven mechanism of *P. aeruginosa* resistance, independent from biofilm formation, could be the aggregation of bacteria that is induced by entropic forces generated by host polymers abundant at chronic infection sites, such as DNA, F-actin or mucin (48).

The survival of bacteria in biofilms is one of the possible mechanisms associated with the inappropriate regulation of neutrophil functions. NETs released by neutrophils represent the source of various biologically active components, which are shown to induce inflammation or tissue damage through multiple mechanisms. NET-derived histones are cytotoxic themselves and were shown to induce epithelial and endothelial cell death (49). While anti-histone antibodies had protective functions, DNase treatment was not effective (49). Moreover, circulating histones can serve as mediators of lung injury during pathological conditions such as trauma, and their levels correlate with lung tissue damage (50). Histones act as damage-associated molecular pattern proteins, activating the immune system and leading to elevated cytotoxicity (51). Possibly, the elevated lung tissue damage observed in WT mice is induced by the high release of histone-containing NETs.

In this manuscript, we show detrimental effect of the elevated IFN signaling during *P. aeruginosa* infection. We observe that *P. aeruginosa* is able to utilize NET components released from activated neutrophils to form biofilms. In the absence of IFN signaling, the release of ROS and NETs is significantly diminished. This leads to reduced biofilm production and lower bacterial count in lungs of infected IFN-deficient mice, as compared to WT. Different bacterial load between WT and IFN-deficient mice is due to altered activation of neutrophils and different amounts of released NETs. Binding to DNA in NETs is a crucial step facilitating biofilm formation by *Pseudomonas*. This could be proven by the infection with *P. aeruginosa* strain that is unable to bind DNA (*pelA* mutant 24480) (37) and therefore cannot efficiently produce biofilms. Bacteria without the protection of biofilms was efficiently cleared in both mouse strains, IFN-deficient and –sufficient. This was independent of the various amounts of NETs measured in these mice, as neutrophil ability to release NETs in response to PA24480 strain was comparable to wild type bacterial strain. It proves the essential role of NETs in the stimulation of biofilm formation.

Multiple treatment modalities are insufficient in infectious complications with *P. aeruginosa*. The reasons for that are on the one hand a high intrinsic heterogeneity of the bacteria, and on the other hand the ability of *Pseudomonas* to form protecting biofilms. Therefore, treatment with factors that disturb biofilm formation should be considered as a strategy to prevent or treat *P. aeruginosa* complications. This is particularly important in inflammatory conditions associated with augmented neutrophil activation. We could show here that NAC, which is a reducing agent and a ROS scavenger, decreases biofilm formation by *Pseudomonas*. Besides its capacity to directly disturb bacterial biofilms by reducing disulfide bonds in mucopolysaccharides (52), NAC has an immunotropic activity by suppressing NETs formation by neutrophils (53). Here, we demonstrated promising therapeutic effect of NAC in *P. aeruginosa* infected mice. NAC treatment significantly improved the clearance of bacteria from lungs, diminished lung tissue damage, and decreased amounts of NETs in lungs. *In vitro* experiments corroborated these results, showing a direct inhibitory effect of NAC on NETs release by neutrophils and, later, on the NET-dependent biofilm production by *Pseudomonas*. In addition, we could validate the efficiency of DNase in the prevention of NET-mediated biofilm formation. This is in agreement with data showing improved clearance of *P. aeruginosa* from the lungs of cystic fibrosis patients after the treatment with DNase (54).

In summary, our work describes detrimental role of type I IFNs during lung infection caused by *P. aeruginosa* in mice. IFNs stimulate pro-inflammatory functions of neutrophils that lead to boosted NETs production by these cells. Binding to NETs activates in turn the formation of biofilms by *P. aeruginosa*. Hidden in biofilms, *Pseudomonas* is able to persist in lungs due to the limited sensitivity to antimicrobials, which causes a progressing therapeutic challenge for patients and clinicians. Therefore, we suggest that NETs-disrupting agents should be considered as a treatment or prophylaxis in clinical conditions associated with elevated type I IFNs levels, such as cancer or viral infections.

## Data Availability

The material is available upon request to interested researchers.

## Ethics Statement

This study was carried out in accordance with the regulatory authorities LANUV (Das Landesamt für Natur, Umwelt und Verbraucherschutz Nordrhein-Westfalen), Germany. Our animal care and used protocols adhere to the regulations of das Deutsche Tierschutzgesetz (TierSchG) and follow FELASA recommendations. The protocol was approved by the regulatory authorities LANUV (Das Landesamt für Natur, Umwelt und Verbraucherschutz Nordrhein-Westfalen).

## Author Contributions

EP: conceptualization, methodology, project administration, formal analysis, writing, and original draft preparation. SB, IS, and AD: formal analysis. VV: formal analysis, software, writing, and original draft preparation. SH: resources, writing, review, and editing. SL: resources, writing, review, and editing. JJ: conceptualization, project administration, supervision, funding acquisition, resources, writing, review, and editing.

### Conflict of Interest Statement

The authors declare that the research was conducted in the absence of any commercial or financial relationships that could be construed as a potential conflict of interest.
