# Supplementary material for: Detrimental Effect of Type I IFNs During Acute Lung Infection With Pseudomonas aeruginosa Is Mediated Through the Stimulation of Neutrophil NETosis
Source: Front Immunol. 2019 Sep 11;10:2190. doi: 10.3389/fimmu.2019.02190 (PMC6749149; doi:10.3389/fimmu.2019.02190)
Supplement: Supplementary file 1 [file Data_Sheet_1.pdf]

Supplementary data

Figure S1

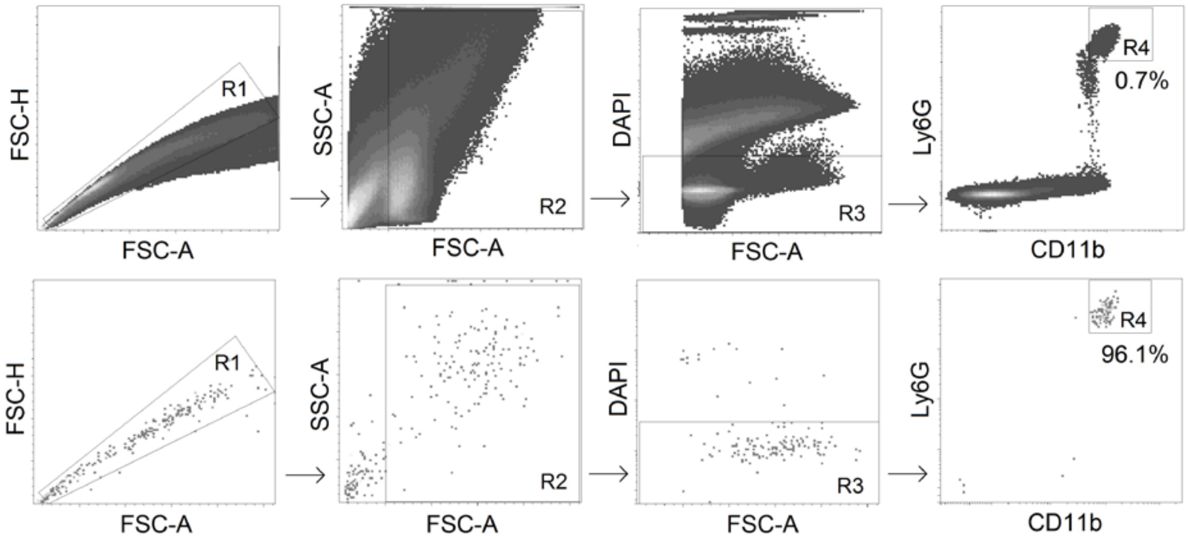

Figure S1. Gating strategy for neutrophils. Single alive CD11b^{+}Ly6G^{hi} neutrophils (R4) were sorted, purity assessment (lower graphs) showed >95% purity.

Figure S2

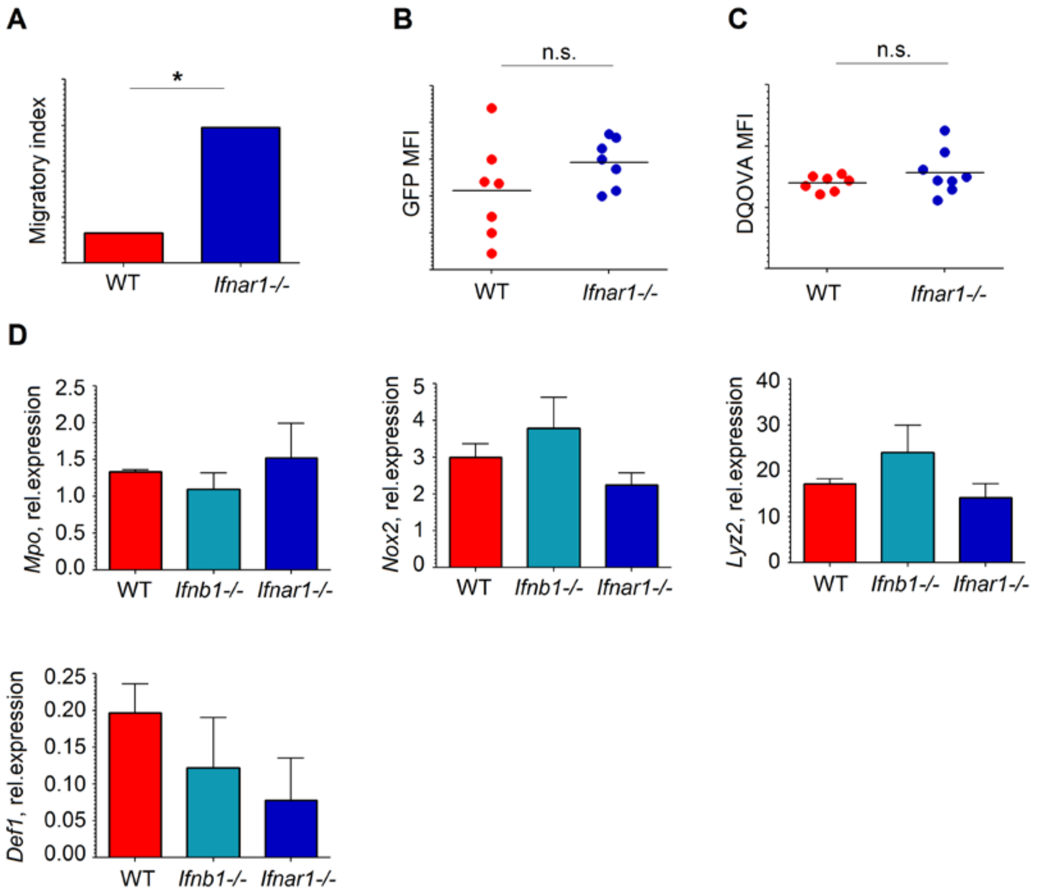

Figure S2. Functional comparison of neutrophils isolated from lungs of WT vs. IFN-deficient mice. **A.** Elevated migratory capacity of *Ifnar1*$^{-/-}$ neutrophils. Lung neutrophils were isolated from *Ifnar1*$^{-/-}$ and WT mice and migration analyzed as described in Material and Methods. **B-D** Similar characteristic of *Ifnar1*$^{-/-}$ and WT neutrophils. Lung neutrophils were isolated from *Ifnar1*$^{-/-}$ and WT mice, and phagocytosis (**B**), endocytosis and phagolysosomal degradation (**C**) analyzed. Data are shown as individual points and mean. Expression of genes involved in antibacterial responses in isolated lung WT vs. *Ifnb1*$^{-/-}$ and *Ifnar1*$^{-/-}$ neutrophils (**D**) was analyzed: *Mpo*, *Nox2*, *Lyz2*, *Def1*, data are shown as mean ± SEM, * $p < 0.05$.
